# Impact of Obesity on Serum Levels of Thyroid Hormones among Euthyroid Saudi Adults

**DOI:** 10.1155/2017/5739806

**Published:** 2017-05-22

**Authors:** Hassan M. Al-Musa

**Affiliations:** Family and Community Medicine Department, College of Medicine, King Khalid University, Abha, Saudi Arabia

## Abstract

**Aim of Study:**

To assess serum thyroid hormones levels among Saudi adults and to correlate participants' serum levels with their grades of body mass index (BMI).

**Methodology:**

A total of 278 adult subjects were recruited. Participants were categorized according to their BMI grades into normal weight (BMI < 25 kg/m^2^), overweight (BMI 25–29.9 kg/m^2^), or obese (BMI ≥ 30 kg/m^2^). Serum thyroid hormones levels were assessed at the central laboratory of Aseer Central Hospital, Abha City, by chemiluminescence immunoassay.

**Results:**

More than three-fourths of participants were either overweight (31.3%) or obese (44.6%). Mean TSH serum levels showed a significantly increasing trend with increasing BMI (*p* < 0.001). A negative trend was observed regarding participants' mean serum levels of fT4 with their BMI, but there were no significant differences in mean serum fT4 levels according to BMI. Moreover, no significant differences were observed in serum fT3 levels according to BMI.

**Conclusions:**

Mean TSH serum levels increase with BMI increase. Further largescale multicentric and longitudinal studies are necessary to prove the association between serum levels of thyroid hormones and BMI of euthyroid adults.

## 1. Introduction

Obesity has become a global epidemic in both developed and developing countries over the last few decades. Approximately 10–15% of all obese people become obese during adolescence [1–3]. In the past 30 years, the prevalence of adolescent obesity has increased by more than 75% [3]. Obesity is associated with psychosocial morbidity and the development of cardiovascular risk factors and diabetes [1].

Obesity represents energy intake from foods exceeding energy expenditure in physical activity [4]. Obesity is now a major problem in the Kingdom of Saudi Arabia. The percentage of overweight and obesity increases with older age groups and this leads to more health problems. In the last three decades, obesity among adults has increased and become a major health problem. In 2003, a study showed that 21% of adults aged 15 years and over were obese/overweight, with an increase from 17% in 1997. Studies in 2000 on the prevalence of overweight and obesity among hypertensive and diabetic adult patients found that 46% of them were obese [5, 6].

Thyroid hormones are known to affect metabolic rate. Recent evidence suggests that thyroid hormones may access the arcuate nucleus and other regions of the hypothalamus to regulate appetite [7]. Thyroid dysfunction can have clinically significant consequences on appetite and body weight. Hypothyroidism classically causes reduced basal energy expenditure with weight gain [8].

Santini et al. [9] reviewed that body weight regulation is accomplished through the fine-tuning between energy intake and energy consumption. Factors determining energy consumption include resting energy expenditure, nonexercise activity, and voluntary physical activity. In man, T3 plays a critical role in temperature homeostasis and is responsible for almost 30% of resting energy expenditure. T3 might also influence resting energy expenditure through regulating spontaneous motor activity.

Experiments on animals have shown a correlation between thyroid hormones and changes in weight [10]. Nevertheless, studies on thyroid hormones in obese adults are inconsistent [11, 12]. Serum thyroid stimulating hormone (TSH) has been reported to be diminished with acute fasting in adults, while serum free T3 (fT3) and free T4 (fT4) and TSH concentrations were reported to be normal in exogenous obesity [13].

Therefore, this study aimed to assess serum thyroid hormones levels among Saudi adults and to correlate serum levels of thyroid hormones with their grades of body mass index.

## 2. Subjects and Methods

This research was conducted during September-December, 2015. It followed a cross-sectional study design. A total of 278 adult subjects (144 males, 51.8% and 134 females, 48.2%) attending a primary health care center in Abha City, Saudi Arabia, were recruited.

The inclusion criteria for participants were being an adult Saudi. The exclusion criteria comprised subjects with any past or present history of thyroid illness, cigarette smokers, patients with chronic liver or renal disease, pregnancy, or taking any drug altering serum TSH levels (e.g., metformin).

A data collection sheet was constructed by the researcher. It included sociodemographic variables (i.e., age, gender), variables related to general examination (i.e., height, weight), and results of laboratory investigations for serum levels of TSH, fT3, and fT4.

Prior to enrollment into the study, all potential participants were informed regarding its objectives and procedures. A written informed consent has been obtained from all participants and they were clearly notified that their participation into this study was completely voluntary.

Weight (in kg) and height (in meters) have been measured while the subjects were barefoot and wearing light clothes. BMI was calculated as body weight (kg) divided by height squared (m^2^). Participants were categorized according to their body mass index (BMI) grades as follows [14]:

- Underweight: <18.5 kg/m^2^.
- Normal weight: 18.5–24.9 kg/m^2^.
- Overweight: BMI of 25–29.9 kg/m^2^.
- Obese: BMI ≥ 30 kg/m^2^.

Phlebotomy was performed after an 8–12-hour overnight fast for free T3, free T4, and TSH measurements. Serum samples were analyzed at the laboratory of Aseer Central Hospital, Abha City, by chemiluminescence immunoassay.

Participants' serum thyroid hormones levels were compared according to their grades of BMI.

The Statistical Package for Social Sciences (SPSS version 22.0) was used for data entry and analysis. Descriptive statistics were calculated and the appropriate tests of significance (i.e., chi square or ANOVA) were applied accordingly. Differences were considered as statistically significant when *p* value was less than 0.05.

## 3. Results

Table 1 shows that participants' age, body mass index, and thyroid hormones serum levels did not differ significantly according to their gender.

None of the participants was underweight. Obesity was significantly higher among female than among male participants (58.2% and 31.9%, resp., *p* < 0.001), as shown in Table 2.

**Figure 1:**
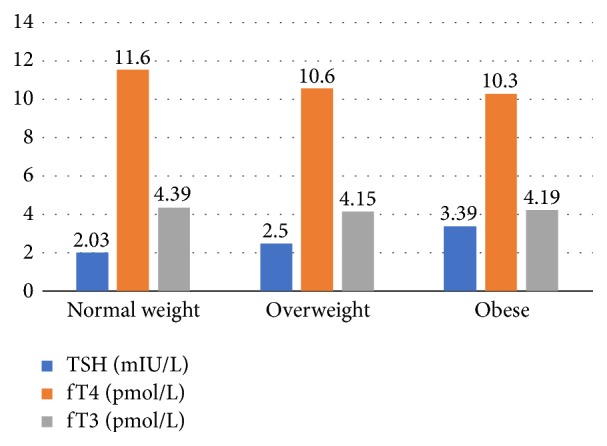
Serum thyroid hormones levels (Mean ± SD) according to grades of body mass index.

Table 3 and Figure 1 show that TSH serum level of normal weight participants was 2.03 ± 1.18 mIU/L, that of overweight participants was 2.50 ± 1.20 mIU/L, and that of obese participants was 3.39 ± 1.18 mIU/L. Mean TSH serum levels showed a significantly increasing trend with increasing BMI (*p* < 0.001). On the other hand, a negative trend was observed regarding participants' mean serum levels of fT4 with their BMI, with highest fT4 levels among participants with normal BMI and lowest among obese participants (11.6 ± 4.1 pmol/L, 10.3 ± 4.5 pmol/L, resp.). However, there were no significant differences in mean serum fT4 levels according to BMI. Moreover, no significant differences were observed in serum fT3 levels according to BMI.

## 4. Discussion

The association between thyroid function and BMI in euthyroid adult individuals has been given a great medical concern [15]. Therefore, the present study aimed to assess serum thyroid hormones levels among Saudi adults and to correlate serum levels of thyroid hormones with their grades of BMI.

Results of the current study showed that more than three-fourths of participants were either overweight (31.3%) or obese (44.6%). Moreover, obesity was significantly higher among female than among male participants.

These findings are in accordance with those of Madani et al. [16], who stressed that, in the Kingdom of Saudi Arabia, obesity has become an important public health problem. Al-Othaimeen et al. [17] stated that prevalence of obesity in Saudi Arabia is 23.6% among women and 14% among men, while prevalence of overweight is 30.7% among men and 28.4% among women. DeNicola et al. [18] noted that, over the past few decades, the Kingdom of Saudi Arabia attained one of the highest prevalence rates for overweight and obesity.

The higher prevalence of obesity among females in Saudi Arabia may be explained by the social restrictions imposed among them that encourage their stay mostly indoors and impose a more sedentary and less active lifestyle among them.

Results of the current study revealed that participants' TSH serum levels showed a significantly increasing trend with increasing BMI. Nevertheless, the significantly increasing trend with BMI was lacking with regard to the fact that fT3 and fT4 did not differ significantly according to participants BMI.

These findings are in accordance with those of Solanki et al. [19], who reported a significant positive association between participants' BMI and their TSH mean serum levels. Moreover, Knudsen et al. [20] indicated that serum TSH is positively correlated with BMI, suggesting a state of possible subclinical hypothyroidism, that is, the presence of raised serum TSH levels despite the presence of serum hormone concentrations within the normal range.

Valyasevi et al. [21] explained the association between TSH and BMI by that TSH may directly stimulate preadipocyte differentiation resulting in adipogenesis. Rotondi et al. [22] added that the impact of bodyweight on thyroid differs according to lower grades of overweight and morbid obesity.

The association between TSH and BMI was explained by Chan et al. [23] to be under the influence of adipose tissue signals and leptin may have significant effects on central regulation of thyroid function through TRH. Zimmermann-Belsing et al. [24] suggested that a positive correlation between serum leptin and TSH also indicates a positive correlation between BMI and TSH.

Several studies revealed that fat cells and precursor forms have receptors for TSH. The signal is transferred with the activation of cAMP-dependent kinase resulting in adipocyte precursor differentiation in adipocytes and lipogenesis [21, 25].

The present study showed no significant associations between male or female participants' serum fT3 or fT4 with their BMI grades.

This lack of significant association between serum thyroid hormones other than TSH with BMI in the present study is in accordance with those reported by some studies, but not with some others. The associations between T3 and T4 with BMI seem to be controversial.

Solanki et al. [19] found that BMI was negatively associated with serum fT4 but had no association with serum fT3 (FT3). Moreover, Roos et al. [26] reported that serum fT4 was negatively associated with BMI. In addition, Iacobellis et al. [27] reported that, in morbidly obese women, lower fT4 values were accompanied by higher BMI values, but no association between BMI and fT3 was found.

In contrast, Manji et al. [8] and Figueroa et al. [28] found no correlation between BMI and serum levels of any of the thyroid hormones in euthyroid individuals. Moreover, Tarim [13] reported that fT3 and fT4 were normal in obese subjects.

Finally, it is to be ascertained that although clear epidemiological associations of serum levels of thyroid hormones with BMI in euthyroid persons have not been completely established, thyroid hormones may constitute an important determinant of the resting energy expenditure in people with normal thyroid function.

However, whether high serum TSH is the cause or the effect cannot be certainly verified. A. Milionis and C. Milionis [15] noted that disorders of the thyroid function may be primary, and the BMI changes may be secondary or vice versa.

Rotondi et al. [29] noted that it is debatable whether obese patients should be diagnosed as having subclinical hypothyroidism based only on their elevated serum TSH levels. It has been suggested that elevated serum TSH might not be enough for diagnosing subclinical hypothyroidism in obese patients. Thus, it is recommended that circulating thyroid antibodies should be measured in obese patients to further support a diagnosis of autoimmune thyroid failure.

In conclusion, mean TSH serum levels increase with BMI increase but other thyroid hormones seem not to be significantly associated with BMI. Further largescale multicentric and longitudinal studies are necessary to prove the association between serum levels of thyroid hormones and body mass index of euthyroid adults.

## Figures and Tables

**Table 1:** Age, body mass index, and thyroid hormones serum levels (mean ± SD) according to participants' gender.

Characteristics	Males (*n* = 144)	Females (*n* = 134)	Total (*n* = 278)	*p* value
Age (years)	33.1 ± 11.1	33.5 ± 9.5	33.3 ± 10.3	0.757
Body mass index (kg/m^2^)	31.5 ± 5.5	32.7 ± 5.6	32.1 ± 5.7	0.070
TSH (mIU/L)	2.82 ± 1.33	2.74 ± 1.29	2.78 ± 1.31	0.632
fT4 (pmol/L)	10.5 ± 4.8	11.5 ± 3.9	11.0 ± 4.1	0.072
fT3 (pmol/L)	4.31 ± 1.68	4.13 ± 1.05	4.22 ± 1.41	0.274

**Table 2:** Grades of body mass index according to participants' gender.

Grades of body mass index	Males (*n* = 144)	Females (*n* = 134)	Total (*n* = 278)
Underweight (<18.5 kg/m^2^)	0 (0.0%)	0 (0.0%)	0 (0.0%)
Normal weight (18.5–24.9 kg/m^2^)	40 (27.8%)	27 (20.1%)	67 (24.1%)
Overweight (25–25.9 kg/m^2^)	58 (40.3%)	29 (21.6%)	87 (31.3%)
Obese (>30 kg/m^2^)	46 (31.9%)	78 (58.2%)	124 (44.6%)

*p* < 0.001.

**Table 3:** Thyroid hormones serum levels (mean ± SD) according to participants' body mass index.

Thyroid hormones	Normal (*n* = 67)	Overweight (*n* = 87)	Obese (*n* = 124)	*p* value
TSH (mIU/L)	2.03 ± 1.18	2.50 ± 1.20	3.39 ± 1.18	<0.001
fT4 (pmol/L)	11.6 ± 4.1	10.6 ± 4.7	10.3 ± 4.5	0.155
fT3 (pmol/L)	4.39 ± 1.47	4.15 ± 1.42	4.19 ± 1.38	0.547

## References

[1] Baur L. A. (2002). Child and adolesecents obesity in the 21st century: an Australian perspective. *Asia Pacific Journal of Clinical Nutrition*.

[2] Zwiauer K. F. (2000). Prevention and treatment of overweight and obesity in children and adolescents. *European Journal of Pediatrics*.

[3] Ackard D. M., Neumark-Sztainer D., Story M., Perry C. (2003). Overeating among adolescents: prevalence and associations with weight-related characteristics and psychological health. *Pediatrics*.

[4] Hill J. O., Wyatt H. R., Reed G. W., Peters J. C. (2003). Obesity and the environment: where do we go from here?. *Science*.

[5] Al-Hazzaa H. M. (2004). Prevalence of physical inactivity in Saudi Arabia: a brief review. *East Mediterr Health J*.

[6] Al-Almaie S. M. (2005). Prevalence of obesity and overweight among Saudi adolescents in Eastern Saudi Arabia. *Saudi Medical Journal*.

[7] Amin A., Dhillo W. S., Murphy K. G. (2011). The central effects of thyroid hormones on appetite. *Journal of Thyroid Research*.

[8] Manji N., Boelaert K., Sheppard M. C., Holder R. L., Gough S. C., Franklyn J. A. (2006). Lack of association between serum TSH or free T4 and body mass index in euthyroid subjects. *Clinical Endocrinology*.

[9] Santini F., Marzullo P., Rotondi M. (2014). Mechanisms in endocrinology: the crosstalk between thyroid gland and adipose tissue: signal integration in health and disease. *European Journal of Endocrinology*.

[10] Koritschoner N. P., Alvarez-Dolado M., Kurz S. M. (2001). Thyroid hormone regulates the obesity gene tub. *EMBO Reports*.

[11] Rosenbaum M., Hirsch J., Murphy E. (2000). Effects of changes in body weight on carbohydrate metabolism, catecholamine excretion, and thyroid function. *The American Journal of Clinical Nutrition*.

[12] Tagliaferri M., Berselli M. E., Calò G. (2001). Subclinical hypothyroidism in obese patients: relation to resting energy expenditure, serum leptin, body composition, and lipid profile. *Obesity Research*.

[13] Tarim O. (2011). Thyroid hormones and growth in health and disease. *Journal of Clinical Research in Pediatric Endocrinology*.

[14] WHO (1998). Obesity: preventing and managing the global epidemic. *Report of a WHO Consultation on Obesity*.

[15] Milionis A., Milionis C. (2013). Correlation between body mass index and thyroid function in euthyroid individuals in Greece. *ISRN Biomarkers*.

[16] Madani K. A., Al-Amoudi N. S., Kumosani T. A. (2000). The state of nutrition in Saudi Arabia. *Nutrition and Health*.

[17] Al-Othaimeen A. I., Al-Nozha M., Osman A. K. (2007). Obesity: an emerging problem in Saudi Arabia. analysis of data from the national nutrition survey. *Eastern Mediterranean Health Journal*.

[18] DeNicola E., Aburizaiza O. S., Siddique A., Khwaja H., Carpenter D. O. (2015). Obesity and public health in the Kingdom of Saudi Arabia. *Reviews on Environmental Health*.

[19] Solanki A., Bansal S., Jindal S., Saxena V., Shukla U. (2013). Relationship of serum thyroid stimulating hormone with body mass index in healthy adults. *Indian Journal of Endocrinology and Metabolism*.

[20] Knudsen N., Laurberg P., Rasmussen L. B. (2005). Small differences in thyroid function may be important for body mass index and the occurrence of obesity in the population. *The Journal of Clinical Endocrinology and Metabolism*.

[21] Valyasevi R. W., Harteneck D. A., Dutton C. M., Bahn R. S. (2002). Stimulation of adipogenesis, peroxisome proliferator-activated receptor-*γ* (PPAR*γ*), and thyrotropin receptor by PPAR*γ* agonist in human orbital preadipocyte fibroblasts. *Journal of Clinical Endocrinology and Metabolism*.

[22] Rotondi M., Leporati P., La Manna A. (2009). Raised serum TSH levels in patients with morbid obesity: is it enough to diagnose subclinical hypothyroidism?. *European Journal of Endocrinology*.

[23] Chan J. L., Heist K., DePaoli A. M., Veldhuis J. D., Mantzoros C. S. (2003). The role of falling leptin levels in the neuroendocrine and metabolic adaptation to short-term starvation in healthy men. *The Journal of Clinical Investigation*.

[24] Zimmermann-Belsing T., Brabant G., Holst J. J., Feldt-Rasmussen U. (2003). Circulating leptin and thyroid dysfunction. *European Journal of Endocrinology*.

[25] Schäffler A., Binart N., Schölmerich J., Büchler C. (2005). Hypothesis paper: Brain talks with fat - Evidence for a hypothalamic-pituitary-adipose axis?. *Neuropeptides*.

[26] Roos A., Bakker S. J. L., Links T. P., Gans R. O. B., Wolffenbuttel B. H. R. (2007). Thyroid function is associated with components of the metabolic syndrome in euthyroid subjects. *Journal of Clinical Endocrinology and Metabolism*.

[27] Iacobellis G., Ribaudo M. C., Zappaterreno A., Iannucci C. V., Leonetti F. (2005). Relationship of thyroid function with body mass index, leptin, insulin sensitivity and adiponectin in euthyroid obese women. *Clinical Endocrinology*.

[28] Figueroa B., Vélez H., Irizarry-Ramírez M. (2008). Association of thyroid-stimulating hormone levels and body mass index in overweight Hispanics in Puerto Rico. *Ethnicity and Disease*.

[29] Rotondi M., Magri F., Chiovato L. (2011). Thyroid and obesity: not a one-way interaction. *Journal of Clinical Endocrinology and Metabolism*.

